# Risky driving behaviour in Abu Dhabi, United Arab Emirates: a cross-sectional, survey-based study

**DOI:** 10.1186/s12889-020-09389-8

**Published:** 2020-08-31

**Authors:** Latifa Mohammad Baynouna AlKetbi, Michal Grivna, Saeed Al Dhaheri

**Affiliations:** 1 Ambulatory Healthcare Services. Abu Dhabi Healthcare Services, 81815 Al Ain, United Arab Emirates; 2 Institute of Public Health, College of Medicine and Health Sciences, United Arab Emirates University, Al Ain, United Arab Emirates; 3 College of Public Health, Abu Dhabi University, Abu Dhabi, United Arab Emirates

**Keywords:** Road traffic collision, driver’s behaviour, Distracted driving, Depression

## Abstract

**Background:**

Traffic collision fatality rates per mile travelled have declined in Abu Dhabi similar to many developed countries. Nevertheless, the rate is still significantly higher than the average of countries with similar GDP and socio-demographic indicators. The literature on the subject in the UAE is limited especially in the area of studying drivers behaviour. This study aims to find determinants of risky driving behaviours that precipitate having a road traffic collision (RTC) in the United Arab Emirates (UAE).

**Methods:**

A cross-sectional, survey-based study was employed. Participants were 327 active drivers who were attending Abu Dhabi Ambulatory Health Care Services clinics. They were provided with a questionnaire consisting of demography, lifestyle history, medical history, driving history, and an RTC history. They were also given a driving behaviour questionnaire, a distracted driving survey, depression screening and anxiety screening.

**Results:**

Novice drivers (less than 25 years old) were 42% of the sample and 79% were less than 35 years. Those who reported a history of an RTC constituted 39.8% of the sample; nearly half (47.1%) did not wear a seatbelt during the collision. High scores in the driving behaviour questionnaire and high distraction scores were evident in the sample. Most distraction-prone individuals were young (90.5% were less than 36 years old). High scores in the driving behaviour questionnaire were also associated with high distraction scores (*p* < 0.001). Respondents with high depression risk were more likely to be involved in the RTC. With each one-point increase in the driver’s distraction score, the likelihood of a car crash being reported increased by 4.9%.

**Conclusion:**

Drivers in the UAE engage in risky behaviours and they are highly distracted. Some behaviours that contribute to severe and even fatal injuries in RTCs include failing to wear a seatbelt and being distracted. Younger people were more likely distracted, while older drivers were more likely to have higher depression scores. Depression is suggested as a determinant factor in risky driving. These findings are informative to other countries of similar socioeconomic status to the UAE and to researchers in this field in general.

## Background

Among all causes of mortality, Road Traffic Collisions (RTC) are the most preventable deaths worldwide. According to the World Health Organization, 3000 people die on the world’s roads every day and several million are injured or disabled each year [1]. In addition, road collisions cost about $600 billion to governments in different economies, a figure that is equivalent to the combined GDP of almost all the developing countries in the world [1]. The U.S. Department of Transportation’s most recent estimate of the annual economic cost of crashes was $242 billion [2]. Fatality rates per population and per mile travelled have declined by half in developed countries since 1975, and the fatality rate per 100 million miles travelled declined from 20.6 in 1975 to 11.4 in 2017 [3].

The UAE compared with industrialized countries, is high in terms of per capita income and has strict laws against alcohol use, strict safety vehicle licensing and high quality of roads [4] and similarly, RTC fatality rate has fallen as well. Nevertheless, the fatality rate is still significantly higher than in countries with similar GBD and socio-demographic indicators [5]. In addition death rate in Abu Dhabi is decreasing and it is lower than that of many developed countries, 18.1 per 100,000 in 2013, still Road Traffic Collision (RTC) is the second leading cause of death. The UAE has a young population with more than one-quarter of its population being younger than 25 years old [6]. The leading cause of death in this age group is road injuries [5]. Therefore, prevention and mitigation of RTCs for younger age groups is especially worthwhile since the youth are in high risk of injury because they are not restrained and have more risky driving practices, driving with no license, drug/alcohol use and distractive driving [7, 8].

Road traffic collisions have not been a research priority in the UAE and in the region despite of the rising awareness of its importance [9, 10]. Therefore, confidence relating to the effectiveness of suggested interventions is uncertain [11]. It is acknowledged that strategies focused on engineering and technological advancements play a role in the prevention and mitigation of RTCs through its effect on drivers’ behaviour. However, direct exploration of human factors in RTCs and targeting the prevention of death and injury by tailored human behavioural interventions and improving judgement is probably of paramount importance.

Driver’s behaviour was targeted in assessment and in interventions because driving skills and bad choices are likely to increase RTC occurrences and result in higher fatality outcomes [11–13]. Although it is known from a study from the UAE that only 2% of vehicle passengers used seatbelts and 13% of motorcyclists wore a helmet, [8] there is a need to study determinants of RTCs and risk taking by the drivers. Many assessment tools exist to screen drivers in order to identify risky drivers. Examples of these tools are the Manchester Driver Behaviour Questionnaire (DBQ) [13] and the Behaviour of Young Novice Drivers Scale (BYNDS) [14].

Implementing interventions in some countries indicated a successful reduction of RTCs [15]. In the US raising the age of licensing eliminated most crashes at age 16 and in Saudi Arabia the introduction of laws on speed restrictions and restrains use have also resulted in positive outcomes [15, 16] . Nevertheless, researchers have suggested that road safety interventions are lacking and that there are shortcomings in the assessments of those performed [16].

This study aimed to identify risky drivers in Abu Dhabi through assessing possible determinants, including driving behaviour using combined validated tools. Although some of these factors maybe studies before but this study aims to study all these factors interaction with deeper inquiry using multiple validated tools. Such results are valuable for targeted intervention design for the Abu Dhabi drivers and could inform other countries as Abu Dhabi have high prevalence of RTC and its population have representation from different countries in the world.

## Method

### Study design

This was a cross-sectional study using self-administered questionnaire. Three hundred and twenty-seven (327) currently licensed drivers participated in the study. Sample size calculation required 317 for a confidence level of 95% and confidence interval of 5.5%. All 18 years or older UAE national participants were and included. They were attending 22 Abu Dhabi government Ambulatory Healthcare (AHS) centres. Fifty questionnaires were given to each of these clinics for distribution to healthy participants attending preventive clinics for premarital university fitness and annual preventive screening. Therefore, the area where the questionnaire was distributed had a greater representation of young participants, which served the purpose of the study because they were the main targets of the research.

### Measures and instruments

Participants reported their demographic data, including: age, gender, occupation, income, education, academic attainment, and living alone or with family. Information on duration of driving history, car type and RTC history was also collected. Additional sections were derived from the below validated surveys. Permissions were obtained to use them from their developers.

a. Driving behaviour assessment: A questionnaire was developed by combining questions from the Manchester Driver Behaviour Questionnaire (DBQ) [10] and the Behaviour of Young Novice Drivers Scale (BYNDS) [14]. The resultant questionnaire was assessed by the investigators and modified. The aim was to select the most relevant factors for the UAE in a shorter version and to avoid questions that were not applicable. This section of the questionnaire included 28 questions and was translated and validated using the front and back translation method. Twenty-eight behaviours were rated on a four-point scale (0 = never, 1 = sometimes, 2 = most of the time, 3 = always). Participants indicated how often they had committed every behaviour in the previous year.

b. The Distracted Driving Survey (DDS): this is a validated 11-item survey which was similarly translated. Answers were reported in a scale ranging from 1 = never to 5 = almost always [17].

c. Mental health assessment: the Patient Health Questionnaire (PHQ-9) and Generalized Anxiety Disorder questionnaire (GAD-7) were used and both are already available in Arabic and are used in all AHS centres as part of routine care.

- Other lifestyle risky behaviours, including smoking, physical inactivity, and dietary habits, were recorded.
- Metabolic parameters, including BMI, lipids, blood pressure, and glycaemic status, were filled by a nurse from the health charts.
- History of health conditions and comorbidities were recorded.

### Data management and statistical analysis

The resulting questionnaire was piloted among 20 family medicine residents for face and content validity and for any mistranslation or interpretation that required resolution. Questionnaires were then distributed by the attending nurse in the preventive clinic. Due to the busy nature of the high-intensity centres no tracking of non-respondents was performed. Consent forms and the questionnaire were distributed for self-completion and participants were assisted when needed by the experienced healthcare personnel who had been briefed about the survey.

Questionnaires were pre-coded for data entry. Data were verified and compared to eliminate key-stroke, range and consistency errors. Analysis was done using the SPSS program, Statistical Package for the Social Sciences (IBM-SPSS version 21, Chicago, Il, USA). Frequencies and cross-tabulations were used to describe the study population. Regression analysis was used to identify significant interactions and predictors of risky driving. Probabilities of less than 0.05 were considered significant.

## Results

Most of the drivers surveyed were younger than 30 years old, and male drivers were driving at a younger age than the females. Novice drivers (less than 25 years old) were 42% of the sample and 79% of them were less than 35 years (See Table 1)

**Table 1:** Demographic characteristics of the study respondents

	Age groups						Percentage within nationality
	<=25	26–35	36–45	46–55	> 55	Total	
**Nationality and Gender**							
UAE male	43 (51.8)	28 (33.7)	10 (12)	2 (2.4)	0	83	78.3
UAE female	11 (47.8)	4 (17.4)	2 (8.7)	5 (21.7)	1 (4.3)	23	21.7
Total	54 (50.9)	32 (30.2)	12 (11.3)	7 (6.6)	1 (0.9)	106	100.0
Non-UAE male	32 (36)	41 (46.1)	14 (15.7)	2 (2.2)	0	89	78.8
Non-UAE female	5 (21.7)	8 (34.8)	9 (39.1)	1 (4.3)	0	23	20.4
Total	38 (33.6)	49 (43.4)	23 (20.4)	3 (2.7)	0	113	100.0
UAE	75 (43.6)	69 (40.1)	24 (14)	4 (2.3)	0	172	78.5
Non-UAE	16 (34.8)	12 (26.1)	11 (23.9)	6 (13)	1 (2.2)	46	21.0
Total	92 (42)	81 (37)	35 (16)	10 (4.6)	1 (0.5)	219	100.0
**Education**							
Elementary	1 (33.3)	0	2 (66.7)	0	0	3	1.3
Secondary	45 (50)	29 (32.2)	12 (13.3)	4 (44)	0	90	40.0
University or higher	48 (37.8)	52 (40.9)	18 (14.2)	7 (5.5)	2 (1.6)	127	56.4
Other	0	2 (40)	3 (60)	0	0	5	2.2
Total	94 (41.8)	83 (36.9)	35 (15.6)	11 (4.9)	2 (0.9)	225	100
**Occupation**							
Student	38 (90.5)	4 (9.5)	0	0	0	42	18.6
Personal business	1 (16.70	3 (50)	1 (16.7)	1 (16.7)	0	6	2.7
Private sector	11 (50)	10 (45.5)	0	0	1 (4.5)	22	9.7
Police or defence	7 (28)	13 (52)	4 (16)	1 (4)	0	25	11.1
Government	18 (22.5)	35 (43.8)	19 (23.8)	7 (8.8)	1 (1.3)	80	35.4
Unemployed	18 (35.3)	19 (37.3)	12 (23.5)	2 (3.9)	0	51	22.6
Total	93 (41.2)	84 (37.20)	36 (15.9)	11 (4.9)	2 (0.9)	226	100
**Income**							
< 5.000 AED	18 (58.1)	9 (29)	3 (9.7)	1 (3.2)	0	31	17.4
5.000–10.000 AED	22 (56.4)	10 (25.6)	5 (12.8)	2 (5.10	0	39	21.9
11.000–20.000 AED	13 (24.5)	26 (49.1)	9 (17)	5 (9.4)	0	53	29.8
> 20.000 AED	13 (23.6)	25 (45.5)	12 (21.8)	3 (5.5)	2 (3.60)	55	30.9
Total	66 (36.9)	70 (39.7)	29 (16.2)	11 (6.1)	2 (1.10)	178	100
**Car type**							
Saloon	42 (41.2)	42 (41.2)	11 (10.8)	5 (4.9)	2 (2)	102	50
Four-wheel	38 (39.6)	31 (32.3)	21 (21.9)	6 (6.3)	0	96	47.1
Truck	0	1 (100)	0	0	0	1	0.5
Other	3 (60)	1 (20)	1 (20)	0	0	5	2.5
Total	83 (40.7)	75 (36.8)	33 (16.2)	11 (5.4)	2 (1)	204	100
**RTC History**							
Yes	32 (36.8)	30 (34.5)	17 (19.5)	7 (8)	1 (1.1)	87	39.7
No	62 (47)	49 (37.1)	16 (12.1)	4 (3)	1 (0.8)	132	60.3
Total	94 (42.9)	79 (36.1)	33 (15.1)	11 (5.0)	2 (0.1)	219	100
**Wear seat belt**							
Yes	13 (31.7)	13 (31.7)	8 (19.5)	6 (14.6)	1 (2.4)	41	47.1
No	23 (50)	14 (30.4)	8 (17.4)	1 (2.2)	0	46	52.9
Total	36 (41.4)	27 (31)	16 (18.4)	7 (8)	1 (1.1)	87	100
**RTC as Driver**							
No	80 (44.1)	64 (35.4)	29 (16)	6 (3.3)	2 (1.1)	181	78.4
Yes	18 (36)	20 (40)	7 (14)	5 (10)	0	50	21.6
Total	98 (42.4)	84 (36.4)	36 (15.6)	11 (4.8)	2 (0.9)	231	100
**Significant RTC as Driver**							
No	90 (43.9)	76 (37.1)	2 (14.1)	9 (4.4)	1 (0.5)	205	88.7
Yes	8 (30.8)	8 (30.8)	7 (26.9)	2 (7.7)	1 (3.8)	26	11.3
Total	98 (42.4)	84 (36.4)	36 (15.6)	11 (4.8)	2 (0.9)	231	100

.

More than half of the participants were college graduates, and 40% had a secondary school certificate. While one-fifth were students, government employees constituted 35% of the respondents. Around one-third had an income of more than 5500$ and another third had an income between 2750$ and 5500$ AED. Half of the respondents drove a four-wheel drive car and the other half owned a sedan car.

**Fig. 1:**
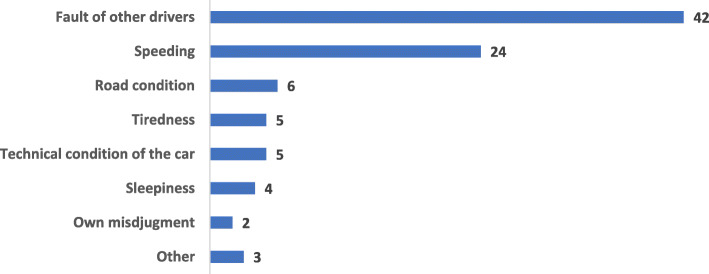
Causes of the RTC reported in the respondent’s opinion

Almost 40% (*n* = 87) of responders reported having a previous history of a RTC and nearly half of them (47.1%) did not wear a seatbelt during their collision. Fifty out of the 87 (57.4%) who reported an previous history of RTC were the drivers during the collision (21.6% of the total responders). More than one in ten (11.3% of the total respondents) reported the collision to be significant, i.e. being transferred to hospital, being admitted to hospital or having medical consequences. Thirty-nine (11.9%) of the respondents reported that a relative had died in an RTC. Figure 1 shows the respondent’s opinion about the causes of the RTC, with other drivers being blamed as the main cause, then speed as the second cause. The least reported cause, from only two respondents, was admitting to their own poor judgement.

**Fig. 2:**
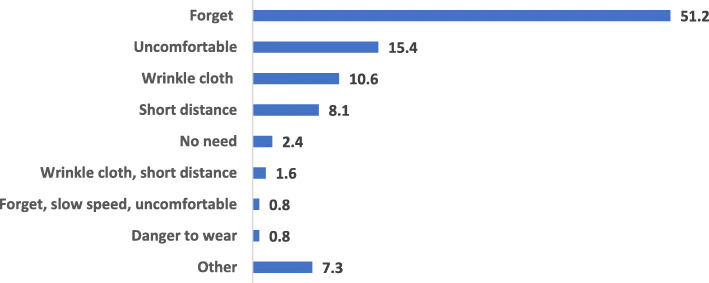
Reasons mentioned by the respondents for not wearing the seatbelt while driving

Reasons for not using a seatbelt are shown in Fig. 2. More than half 51.2% (*n* = 63) of subjects reported that the main reason for not wearing a seatbelt is that they forgot, 15.4% (*n* = 19) thought that it is uncomfortable to wear, while 10.6% (*n* = 13) of the total respondents chose not to wear a seatbelt because it wrinkles their clothes.

Table 2 shows the distribution of the four main survey parts; Drivers’ Behaviour survey, Drivers’ Distraction survey, PHQ-9 and D. GAD-7. Responses to the 28 questions from the Driving Behaviour Questionnaire (DBQ) are grouped by the type of the violation and Table 3 shows the responses to individual questions. The surveyed participants show prevalent risky behaviour more in ordinary violations, such as their passengers not wearing a seatbelt, speeding to catch an event, speeding when a traffic light is yellow, speeding when there is no radar or camera present, and did not wear a seatbelt in a short distance. The proportion of participants who reported never engaging in these behaviours ranged from 41.3% (not wearing a seatbelt) to 49.2% (didn’t wear a seatbelt in a short distance). Risky violations that are less prevalent are drifting, driving without a license, driving under the influence of alcohol or an illicit drug (between 79.3 and 88.5% of the respondents reporting never having engaged in these behaviours). Regarding lapses and errors, around one-third (32.5%) did misread signs, 27.3% failed to notice pedestrians crossing, and 38.5% misjudged the speed of an oncoming vehicle. On the other hand, driving when feeling sleepy was a risky behaviour that is considerably prevalent, with 47.3% reporting having done it. Aggressive violations, such as racing away from a traffic light with the intention of beating the driver next to them and sounding a horn to indicate annoyance to another road user, was reported by 18.6 and 23.3% of respondents, respectively.

**Table 2:** Distribution of the four main survey parts. A. Drivers’ Behaviour survey, B. Drivers’ Distraction survey, C. PHQ-9 and D. GAD-7

**A. Drivers’ Behaviour Questionnaire average score grouped by the type of the violation**						Mean	Standard Deviation
Errors and lapses						0.34	0.43
Ordinary violations (speed)						0.56	0.56
Ordinary violations (traffic lights)						0.47	0.51
Violations (not wearing seatbelt)						0.79	0.74
Violations (alcohol related)						0.15	0.39
Aggressive violations						0.58	0.72
Situational violations (rain, sleep, mood)						0.68	0.63
All violations						0.51	0.39
Total DBQ score mean						11.36	9.58
**C. PHQ-9**							
Low risk						138	57.3
Mild risk						78	32.4
Moderate risk						20	8.3
Moderately severe risk						4	1.7
Severe risk						1	.4
**D. GAD-7**							
Low risk						184	75.1
Mild risk						53	21.6
Moderate risk						8	3.3
**B. Distraction prone or averse**							
Age group	<=25 N (%)	26–35 N (%)	36–45 N (%)	46–55 N (%)	> 55 N (%)	Total N (%)	
Distraction-averse	33 (34)	33 (34)	24 (24.7)	5 (5.2)	2 (2.1)	97 (44.3)	
Distraction-prone	60 (49.2)	48 (39.3)	9 (7.4)	5 (4.1)	0	122 (55.7)	
Total	93 (42.5)	81 (37)	33 (15)	10 (4.5)	2 (0.90)	219 (100)

**Table 3:** Reponses to the Drivers’ Behaviour Questionnaire’s questions

Questions	Mean	SD	Never (%)	Sometimes (%)	Most of the time (%)	Always (%)
You drove after taking Alcohol	0.87	0.87	88.5	9.5	1.4	0.7
You drove after taking an illicit drug such as marijuana or ecstasy	0.87	0.91	87.9	10	1.7	0.3
You carried more passengers than could legally fit in your car	0.79	0.82	85.7	12.3	1.4	0.7
Race away from traffic lights with the intention of beating the driver next to you	0.71	0.83	81.4	16.9	1.3	0.4
Do you participate in drifting on the normal road	0.66	0.79	79.3	17.3	2.4	1
Disregard the speed limit on a residential road/city/highway	0.65	0.73	78.9	16.9	3.4	0.8
You drove without a valid license because you hadn’t applied for one yet or it had been suspended	0.65	0..68	78.3	18.3	2.4	1
Sound your horn to indicate your annoyance to another road user	0.41	0.63	76.7	20	2.4	0.8
Hit something when reversing that you had not previously seen	0.60	0.73	75.1	22.7	1.7	0.4
Queuing to turn left onto a main road, you nearly hit the car in front of you	0.57	0.73	74.7	21.8	2.6	0.9
Fail to notice that pedestrians are crossing when turning into a side street from a main road	0.58	0.72	73.7	24.6	0.8	0.8
You did illegal U-turn	0.52	0.71	72.4	24.1	2.7	0.7
Get into the wrong lane approaching a roundabout or a junction	0.47	0.68	71.9	24.3	3	0.9
Misread the signs and exit from a roundabout on the wrong road	0.45	0.63	67.5	29.4	2.2	0.9
Does your over speed changed (stopped speeding) with any fine you paid	0.43	0.71	67.1	24.3	6.5	2.1
You misjudged the speed of an oncoming vehicle	0.36	0.57	61.5	32.3	5.5	0.7
You turned right into the path of another	0.33	0.58	60.8	33.4	3.4	2.4
Forget where you left your car in a carpark	0.32	0.56	59	32.1	7.3	1.7
You drove faster if you were in a bad mood	0.30	0.56	56.2	32.9	9.2	1.7
If there was no red light camera, you drove through intersections on a red light	0.29	0.52	53	38.2	6.4	2.4
You drove when you knew you were feeling sleepy	0.27	0.51	52.7	36.1	9.5	1.7
Do you rush to catch an event	0.25	0.55	50.2	37.1	9.3	3.4
You didn’t wear a seatbelt if it was only for a short	0.21	0.47	49.2	33.7	13.8	3.4
You drove over the speed limit in areas where it was unlikely there was a radar or speed camera	0.26	0.56	47.1	44.1	5.8	3.1
You speed up when the lights went yellow	0.26	0.55	45	47	6.4	1.7
Do you rush to be on time for work or school	0.17	0.46	42	41.7	11.9	4.4
Your passengers didn’t wear seatbelts	0.14	0.42	41.3	38.6	12.4	7.7
You drove in the rain	0.14	0.44	39.9	38.5	16.2	5.4
DBQ Average score	0.27	0.55	54.03	35.89	7.76	2.36

**Fig. 3:**
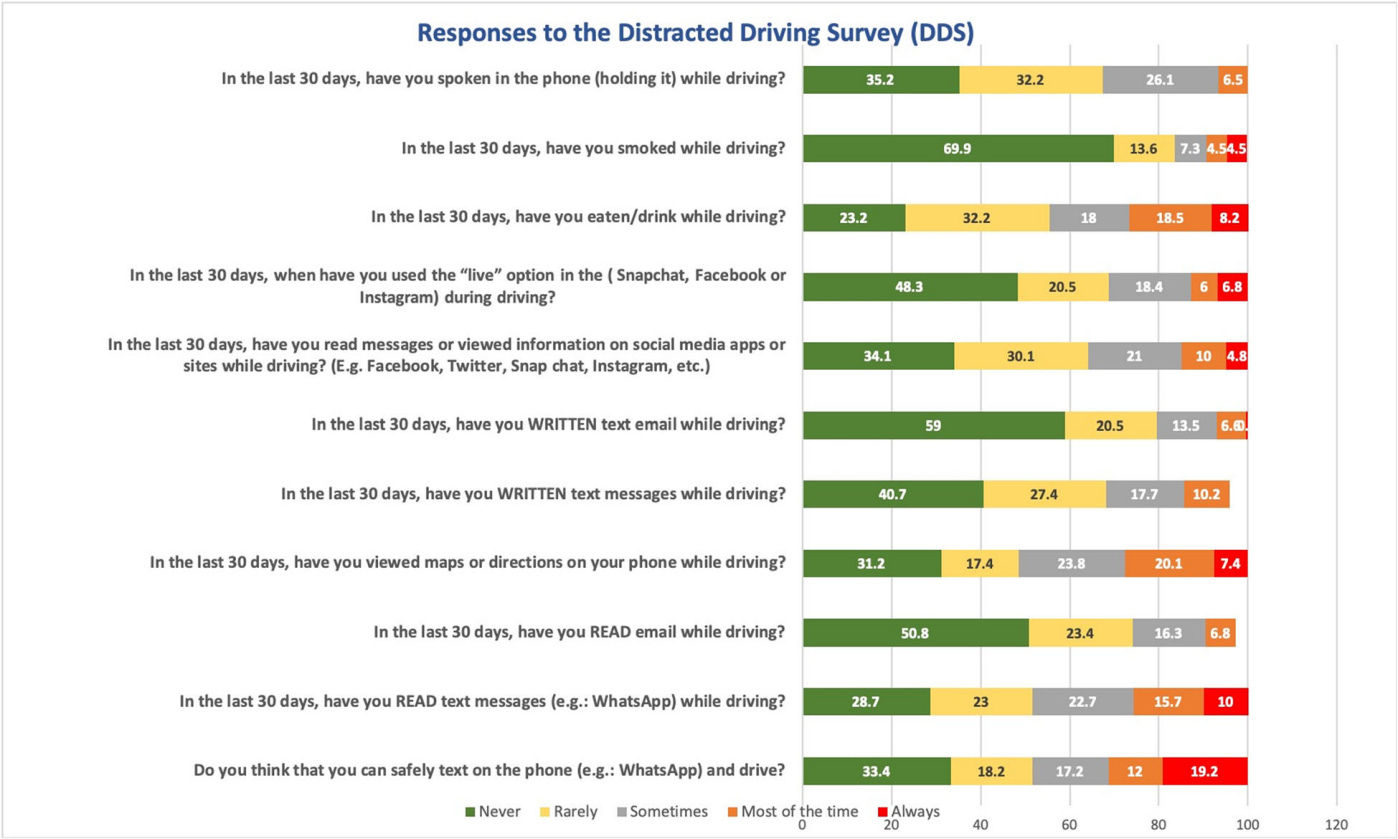
Responses to the Distracted Driving Survey (DDS)

Distracted behaviour was even more prevalent (Fig. 3). Responses to the Distracted Drivers Survey (DDS) shows that 55.7% of the respondents are distraction prone (distraction score less than 12) and 44.3% distraction averse (distraction score more than 11). One in five (19.4%) have a distraction score of 20 or more (Table 2). The most distraction prone group is younger people (90.5% are less than 36 years old). With regression analysis, a high DBQ score is associated with a high distraction score (*p* < 0.001). Age, on the other hand, is the other associated factor with the distraction score but it has an inverse relationship: the younger the responder, the higher the distraction score (*p* = 0.003). The most prevalent distractions were talking on the phone, reading messages, viewing maps, reading text messages, or eating or drinking, with a range from 23.2 to 35.2% never having engaged in these activities.

The main purpose of this study was to predict important associations between studied variables for risky drivers and these were identified using regression analysis. Higher scores from the DBQ were associated with higher PHQ-9 scores (*p* < 0.0001), more consideration of placing financial fines (< 0.0001), use of live recording social media when driving (*p* = 0.003), smoking while driving (*p* = 0.026), and reading WhatsApp while driving (p < 0.0001).

**Fig. 4:**
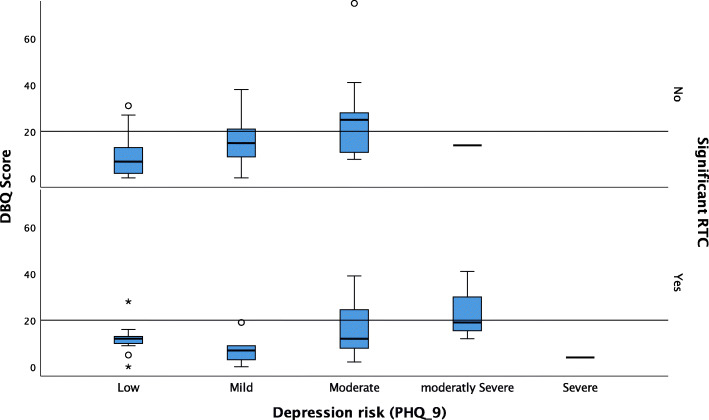
Risk of depression (PHQ-9 score category) related to Drivers’ Behaviour Questionnaire score and the history of significant RTC

Distracted drivers, as a risk factor for RTC, are more likely to be younger (*p* < 0.0001), eat out more frequently (*p* = 0.021), have violations related to speeding (*p* = 0.001), have driven with no license (*p* = 0.008), or have driven in bad mood (*p* = 0.038). On the other hand, participants reporting a higher risk of depression are more likely to be older (*p* = 0.028), with a history of significant RTCs (*p* < 0.0001), with a higher GAD-7 score (p < 0.0001), and a higher Drivers Behaviour Questionnaire score (p < 0.0001). Figure 4 shows these associations.

Self-reported RTC was significantly associated with high PHQ-9 scores (*p* < 0.001 OR is 1.144 (1.056–1.240), age, *p* = 0.023 OR is 1.051 (1.006–1.098)) and a high distraction score (*p* = 0.018 OR is 1.049 (1.008–1.091)). For each one-point increase in the PHQ-9 score, the risk of an RTC increased by 4.4%. With each one-point increase in the distracted drivers’ score (which is reported on a 0–44 scale with 44 being highest risk behaviours), the likelihood of reporting a car crash increased by 4.9%, and with each additional year in age the RTC risk increased by 5.1%. See Table 4.

**Table 4:** Determinants of A. Drivers’ Behaviour Questionnaire (DBQ), B. Distraction Score, C. Patient Health Questionnaire (PHQ-9) Score using regression

	B	P -value	95.0% CI	
**A. Driver Behaviour Questionnaire DBQ**				
PHQ 9 Score	0.493	0	0.287	0.699
Changes behaviour after financial fines	5.418	0	4.148	6.687
Using snap while driving	1.38	0.003	0.47	2.289
Smoking while driving	1.013	0.026	0.125	1.901
Reading WhatsApp while driving	1.543	0	0.745	2.342
**B. Distraction Score**				
Age	−0.21	0.021	−0.38	−0.03
Eating out frequently	1.261	0.033	0.101	2.42
Violations Speeding	4.827	0.001	1.955	7.699
Driving without license	3.285	0.008	0.854	5.716
Driving in bad mood	2.264	0.038	0.131	4.398
**C. PHQ-9**				
History of being the driver in a RTC	1.09	0.028	0.119	2.06
Age	0.047	0.057	−0.001	0.096
History of being the driver in a RTC that results in Significant impact as major injury or admission.	2.987	0	1.672	4.302
GAD-7 Score	0.715	0	0.591	0.838
DBQ Score	0.115	0	0.074	0.157

None of the other variables surveyed, including gender, occupation, income, education, nationality, BMI, physical activity, smoking, medical history of any chronic illness years since starting to drive and using seatbelt, showed any significant association with self-reported RTC.

## Discussion

This study validated a tool to assess driving behaviour among adults in Abu Dhabi Emirate, United Arab Emirates. The tool has proved to be useful to describe the current situation and to provide advice regarding important predictors of risk-taking while driving. The questions from the Manchester Driver Behaviour Questionnaire (DBQ) [13] and the Behaviour of Young Novice Drivers Scale (BYNDS) [14] were used in many countries, including the United Arab Emirates and Qatar [12, 18]. The cross-country comparisons of the DBQ concluded that the scores can be compared with a confidence [19]. Important additions are areas of growing interest: impact on drivers, mental health and driving distractions [20].

The participants surveyed in this study admitted fewer of the listed behaviours than did those from Britain, Finland or the Netherlands [12]. Violations were more prevalent in Europe, while in Abu Dhabi non-use of seatbelts and other ordinary violation, such as speeding to catch an event or to get to work, were more common. Less prevalent behaviours among Abu Dhabi drivers were aggressive behaviour and driving under the influence of alcohol.

The prevalence of self-reported RTC (39.8%) is high especially, that half of participants were the drivers. The opinion what caused the RTC shows an alarming picture, although it cannot be considered to be the true reflection of reality. It was rare for the respondents to identify the fault in the RTC to be the reporting driver. The tool also identified risky choices taken by the study participants while driving. Regarding the prediction of an RTC in this study, those reporting more violations related to aggressive behaviour, driving sleepy or under rainy conditions, driving in bad mood or having a high.

PHQ-9 score were more likely to report an RTC than those with no RTC history. The environmental factors and road designs are playing an important role in traffic safety [21, 22] The attention of the driver is important in order to see road defects or anomalies in the road design and appropriately react at the urban intersections [21, 22]. Although years since the start of driving was not significant with having an RTC history, age was such a factor.

Significantly, experience with the RTC is greater among those reporting certain behaviours, such as traffic light violations or driving under the influence of alcohol. Most importantly, violations and lapses predicted self-reported RTCs. Although many studies found that DBQ was a prominent measurement scale to examine drivers’ self-reported aberrant behaviours, some experts have argued against this conclusion and that using only self-reported data is not reliable [23, 24]. It is possible that underreporting is high due to recall bias. A recent study reached a similar conclusion, namely that methodological factors and dissemination bias have inflated the published effect sizes of the DBQ and that a greater level of care should be taken if the DBQ continues to be used in traffic safety research [25]. A study of registered RTCs, not self reported, in correlation to the tool used in this study, DBQ, is needed in the UAE to assess the tool as a correct predictor of RTC.

The low rate of seatbelt usage and reasons for not wearing a seatbelt suggest a possible lack of insights regarding potential fatal consequences. Being forgetful and feeling uncomfortable are the most common reasons given for not using a seatbelt, but one in ten stated that it is due to wrinkling their clothes. This necessitates appropriate measures to increase community awareness. With 61% reporting seatbelt usage always or most of the time in this study, this is a considerably lower figure compared to seatbelt use in the US, where 94% of drivers wear a seatbelt [26]. On the other hand, this score is higher than others reported in the region, such as in Saudi Arabia (34%). Additionally, around half of those involved in an RTC in this study were not wearing a seatbelt at the time of the RTC [27]. A recent study in the US regarding seatbelt use by drivers involved in fatal RTCs reported that half of them were not wearing a seatbelt. Compared to belted drivers, unbelted drivers were over four times more likely to die in an RTC [26].

Regarding the drivers’ mental health and its association with making risky choices, this consideration emerged as an area needing further research and interventions. With one out of ten of the respondents having a risk of mild to severe depression, this number is similar to reported prevalence in the UAE, between 12.5 and 28.6% [28]. Also, those with high PHQ-9 scores reported having more significant RTCs and risky driving behaviours. These findings raise the question of whether mental status would be the cause or the result of the RTC. Evidence is growing that has examined rates of RTCs and traffic violations for drivers with mental illnesses, while other studies have examined the performance of people with mental illnesses on driving simulators [29]. Drivers who reported feeling depressed were more than twice as likely to be at fault for their collision than drivers who did not report such feelings. Additionally, depression was associated with increased aggressive and risky driving behaviour. The epidemiological evidence is increasingly showing that depression may have a detrimental impact on collision risk [30]. In a study assessing 208 inpatient psychiatric patients in the areas of reaction time, anticipation of speed, coordination, decision-making skills, and risk-taking, only 33 had scores compatible with the requirements to obtain a driver’s license, and 84% failed at least one of the required tests. Of patients with a driver’s license who drive almost every day, 79.5% registered scores that would not allow them to obtain or renew their licenses [31]. In this study, risk of depression was a determinant factor that requires future research and consideration by traffic safety authorities.

Finally, it was alarming to find the high distraction scores in this sample. Bergmark et al. [17] found that texting “always” while driving was a response of 7% in their study, compared to 19.2% in this sample. Viewing maps while driving was 4.8% in their study, compared to 7.4% in this study. Those who always read text messages while driving were 2.2% in their study, compared to 10% in this study. All indicating higher rates among respondents of this study. Finally, those who read messages or viewed information on social media apps or sites while driving was 2.2% in their study, compared to double the rate in this study, 4.8%. All of these factors, in addition to highly prevalent distractions such as talking on the phone or eating or drinking while driving, reflect a risky behaviour that necessitates immediate and firm multifaced interventions.

In a recent study in Saudi Arabia using a mobile phone was associated with higher severity and prevalence of disability rates and was associated with a 44% greater likelihood of incurring a severe RTC [32]. Worldwide figures on the impact of distraction while driving to RTC ranges from 8% in the Netherlands, 11% in the US, to 37% in Spain [33]. Compared to the study by Bergmark et al. [17], which is similar to this study, a significant association was found between DDS and an RTC history. In their study, for every single unit increase of the DDS score, the likelihood of reporting a car crash increases by 7%, compared to 4.9% in this study.

There are couple of limitations in our study. The results were based on the convenient sample so it is difficult to draw conclusions that can be generalized to the whole UAE population. Nevertheless, it highlights the risky driving behaviour, especially among young drivers in the UAE using validated tools. Although self-reporting of behaviour has been mentioned as having a risk of recall and reporting bias, there is sufficient evidence from the literature on the DBQ if there is similar risk of bias [23, 24] and in reporting about other behaviours, such as seatbelt usage, that self-reporting of behaviour represents an efficient alternative to observational studies for tracking changes in actual behaviour [34].

## Conclusions

Drivers’ behaviour in the UAE is risky, drivers are highly distracted, and depression must be considered as an important factor in risky driving. While there have been numerous technological advancements in vehicular and environmental safety, human behaviours, as distracted driving or not-use of restraints frequently contribute to severe injuries and fatalities in RTCs. Younger people were more likely to be distracted, while Depression is suggested as a determinant factor in risky driving. The concluded risk factors for RTC, including distractibility and risky behaviour may not be only specific to the study social and environmental conditions in the UAE. The study’s results may be applied in other countries, especially that UAE have large number of nationalities and there was no difference found in determinants of behaviour between UAE and non-UAE populations. Nevertheless, this is to be confirmed by replicating the study elsewhere.

## Data Availability

The datasets used and/or analysed during the current study available from the corresponding author on reasonable request.

## Abbreviations

UAE: United Arab Emirates
GDP: Gross Domestic Product
RTC: Road Traffic Collision DBQ Manchester Driver Behaviour Questionnaire
BYNDS: Behaviour of Young Novice Drivers Scale
PHQ9: Patient Health Questionnaire
GAD7: Generalised Anxiety Disorder
AED: UAE Dirham (currency)
